# Commentary on training and education in medical statistics, in celebration of 40 years of statistics in medicine and 50 years of the MSc medical statistics at LSHTM

**DOI:** 10.1002/sim.9292

**Published:** 2022-02-23

**Authors:** Deborah Ashby

**Affiliations:** ^1^ School of Public Health Imperial College London London UK

Forty years ago, I was an MSc Medical Statistics student at LSHTM, and a very new member of the Royal Statistical Society. In 1981, in the medical world, cases of a new disease, later termed AIDS, were being talked about and the HIV virus was as yet unknown. Meanwhile, in my world, I learned about classical statistical inference with brief mention of something called Bayes, epidemiological study designs, and analytical techniques, with statistical analyses done by typing code line by line on to punchcards, which were then deposited to be run, with the output available for collection some while later. Reading a journal article meant going to a library and getting a heavy tome out onto a desk. And a new journal to be called *Statistics in Medicine* was being planned.

For me, my time as am MSc student was the beginning of long associations with LSHTM, the Royal Statistical Society, and Statistics in Medicine. I attended Royal Statistical Society meetings, often conveniently held at LSHTM in those days. I got involved in the Medical Section Committee, was elected to Council, and most recently have had the great honor to serve as President. When the Medical Section ran a joint meeting with London School of Economics on Methodological and Ethical Issues in Clinical Trials featuring the classical/Bayes debate inter alia I edited a Special Issue of Statistics in Medicine to publish those proceedings.^1^ It was the first time I met Don Berry, who along with David Spiegelhalter, had a profound influence on my thinking as to the role of Bayes in trials, although at that stage it was not yet happening in real time. I have subsequently published a number of papers in Statistics in Medicine on aspects of Bayesian trials, including a tutorial on Bayesian data monitoring^2^ and was invited to review Bayesian Statistics in Medicine for the 25th anniversary volume.^3^ At LSHTM I initially visited for collaborations when my PhD supervisor Stuart Pocock, had moved there, and later was invited back to teach Bayesian inference on the MSc for several years running—a lovely illustration of how the MSc prepared me to think and be open to new ideas, which I was then able to take back to challenge future generations of students.

In those intervening years, the world has changed—we can now do large, complex analyses using small portable devices, using numerical simulation techniques which have revolutionized the ways we can use Bayesian approaches. Those same devices can access any journal article instantaneously and enable meetings with people the other side of the globe. The HIV virus was identified in 1983^4^ paving the way for treatments to be developed to transform it from a fatal disease into a disease with which one lives. Trials and other studies have played a pivotal role, and those trials have influenced the way we do research, although we do not yet, 40 years on, have a vaccine. HIV was not mentioned on my MSc, but the education prepared statisticians to be able to step up to the challenge.

I titled my Royal Statistical Society Presidential Address “Pigeonholes and mustard seeds: growing capacity to use data for society” with a key theme being how we develop people with the skills needed at the scale needed for today's challenges.^5^ The mustard seeds refer to Florence Nightingale's quote “I never lose an opportunity of urging a practical beginning, however small, for it is wonderful how often in such matters the mustard‐seed germinates and roots itself” and as an example I cited Peter Armitage foresight in setting up the MSc in Medical Statistics at LSHTM. There were just five students in the first cohort, and I was later taught by one of those, Richard Hayes, so they were indeed the mustard seeds for subsequent cohorts which now number many hundreds, if not thousands, in all corners of the globe.

In my Presidential Address, I talked about both the greater depth of training needed for some individuals, as well as a hugely greater increase in the numbers of people needed, from those in the traditional statistical and mathematical moulds as well as data scientists and those who use need to use data science skills intelligently as a part of their work. I said that those who work in AI and ML need to understand statistical principles, but it is equally important that statisticians should learn more about AI and ML, both for their own sakes, and to be able to engage with those communities. I also said that part of the solution is more flexible models of education and training. As well as traditional campus‐based courses, there is an explosion in on‐line learning, ranging from individual massive open on‐line courses, which are often available free or at minimal costs, to full Master's courses, which are well suited to areas such as ours. I also predicted that, for some, these will replace traditional campus‐based study, but for others they can be a source of accessible continuing professional development.

Finally, I said that different people may need different levels of various aspects at different career stages, but it should also be possible to transition between them when needed. That can have profound implications for how we think about education and training. Personally, I think of statistics as a language for communicating what we can learn from data. I suggested that the Common European Framework of Reference for Languages,^6^ may be a helpful analogy in planning training and continuing professional development for individuals and groups. Explicitly learning how to work in “team mode” is also a priority, with communication being a critical skill. On communication, the Royal Statistical Society has also had a number of initiatives to offer training and to work in other ways with journalists and politicians to increase the quality of statistical discourse in society. Although that may still be at the mustard seed stage, I trust those mustard seeds will germinate.

Today, in 2021, MSc students are studying in extraordinary circumstances due to COVID‐19, and on‐line learning has evolved faster than any of us could have predicted in 2019. But on the bright side, what took 40 years with HIV has been achieved in a small fraction of that time for COVID‐19, from the identification of the pathogen SARS‐COV‐2 and a growing understanding of the epidemiology, to the setting up of platform trials for testing of treatments and the development and rollout of vaccines within a year. I have never considered myself primarily an infectious disease researcher, but like many others, have had to learn fast. So I find myself involved in the REACT studies monitoring the prevalence of the virus in England,^7^, ^8^ which combines design principles I learnt at LSHTM with modern analyses to exploit the temporal and spatial structure of the data. I also play a role as chair of the DSMC for the PRINCIPLE trial, an adaptive platform trial to look at treatments for patients with COVID‐19 in the community (https://www.principletrial.org), which uses Bayesian design and analysis to make most efficient use of the data. Coming full circle, Berry Consultants, founded by Don Berry with Scott Berry to meet the growing demand for Bayesian approaches in trials, are partners in this. Both REACT and PRINCIPLE are large‐scale initiatives and were set up in a tiny fraction of the time it normally takes to get research projects underway. The pace of these and similar projects is an extraordinary testament to science and scientists, including statisticians‐ and to those who trained those scientists and statisticians. Prosecuting researching in a novel area at this pace relies on really firm foundations.

Many of today's students will spend careers working though the short and long‐term consequences of COVID‐19 on health, and other health problems have not gone away. And who knows what else may emerge to challenge us. May LSTHM and other institutions continue to educate and train the future generations of statisticians, and Statistics in Medicine continue to publish the fruits of those labors and contribute to further understanding and development of statistics that will improve the health of populations everywhere.
